# Thioredoxin Domain Containing 5 (TXNDC5): Friend or Foe?

**DOI:** 10.3390/cimb46040197

**Published:** 2024-04-04

**Authors:** Seyed Hesamoddin Bidooki, María A. Navarro, Susana C. M. Fernandes, Jesus Osada

**Affiliations:** 1 Departamento de Bioquímica y Biología Molecular y Celular, Facultad de Veterinaria, Instituto de Investigación Sanitaria de Aragón, Universidad de Zaragoza, E-50013 Zaragoza, Spain; h.bidooki94@gmail.com (S.H.B.); angelesn@unizar.es (M.A.N.); 2 Centre National de la Recherche Scientifique (CNRS), Institute of Analytical Sciences and Physico-Chemistry for Environment and Materials (IPREM), Universite de Pau et des Pays de l’Adour, E2S UPPA, 64 000 Pau, France; susana.fernandes@univ-pau.fr; 3 MANTA—Marine Materials Research Group, Universite de Pau et des Pays de l’Adour, E2S UPPA, 64 600 Anglet, France; 4 Instituto Agroalimentario de Aragón, CITA-Universidad de Zaragoza, E-50013 Zaragoza, Spain; 5 Centro de Investigación Biomédica en Red de Fisiopatología de la Obesidad y Nutrición (CIBEROBN), Instituto de Salud Carlos III, E-28029 Madrid, Spain

**Keywords:** TXNDC5, ERp46, thioredoxin enzymes, friend, foe, endoplasmic reticulum, dual role, cancer, diabetes, heart diseases, infection

## Abstract

This review focuses on the thioredoxin domain containing 5 (TXNDC5), also known as endoplasmic reticulum protein 46 (ERp46), a member of the protein disulfide isomerase (PDI) family with a dual role in multiple diseases. TXNDC5 is highly expressed in endothelial cells, fibroblasts, pancreatic β-cells, liver cells, and hypoxic tissues, such as cancer endothelial cells and atherosclerotic plaques. TXNDC5 plays a crucial role in regulating cell proliferation, apoptosis, migration, and antioxidative stress. Its potential significance in cancer warrants further investigation, given the altered and highly adaptable metabolism of tumor cells. It has been reported that both high and low levels of TXNDC5 expression are associated with multiple diseases, such as arthritis, cancer, diabetes, brain diseases, and infections, as well as worse prognoses. TXNDC5 has been attributed to both oncogenic and tumor-suppressive features. It has been concluded that in cancer, TXNDC5 acts as a foe and responds to metabolic and cellular stress signals to promote the survival of tumor cells against apoptosis. Conversely, in normal cells, TXNDC5 acts as a friend to safeguard cells against oxidative and endoplasmic reticulum stress. Therefore, TXNDC5 could serve as a viable biomarker or even a potential pharmacological target.

## 1. Introduction

Thioredoxin domain-containing 5 (TXNDC5) is a member of the protein disulfide isomerase (PDI) family. It is also known as endoplasmic reticulum protein 46 (ERp46), endothelial protein disulfide isomerase (EndoPDI), or protein disulfide isomerase family A member 15 (PDIA15) [1,2]. TXNDC5 is highly expressed in endothelial cells (ECs), fibroblasts [1], pancreatic β-cells [3], liver cells, and hypoxic tissues, such as cancer endothelial cells and atherosclerotic plaques [2]. It is mainly expressed in the endoplasmic reticulum (ER), with additional expression in the cytoplasm [2]. TXNDC5 is an ER-localized protein disulfide isomerase that functions to facilitate proper protein folding, prevent unfolded protein response (UPR)-related apoptosis [1,3], oxidative folding of some secretory proteins, to modulate adiponectin signaling, insulin production [3], contribute to the stability of extracellular matrix (ECM) proteins [4] and mannose trimming activity for glycoprotein substrates [5].

Furthermore, TXNDC5 is crucial in regulating cell proliferation, apoptosis, migration, and antioxidative stress [2]. TXNDC5 has three redox-active Trx-like domains, each with a CGHC active site that catalyzes the formation of native disulfide bonds and rearranges the disulfide bonds [1,3,6] to switch off the oxidative activity [3]. TXNDC5 is a potent disulfide initiator, particularly in the early stages of translation. Disulfide bonds are introduced into the nascent polypeptide outside the ribosome exit site through a dithiol–disulfide exchange reaction with the assistance of oxidoreductases, including TXNDC5 and other members of the PDI family [7,8]. The abnormal expression of TXNDC5 and single nucleotide polymorphisms (SNPs) in the TXNDC5 gene have been found to be associated with the risk of various diseases, including organ fibrosis (affecting approximately 25% of the global population) [9], atherosclerosis (causes 17.9 million deaths per year) [10], diabetes (affects approximately 463 million adults globally) [11], liver disease (causes over 2 million deaths annually worldwide) [12,13], rheumatoid arthritis (RA) (affects 0.24% of the global population) [14], cancer (an estimated 19.3 million new cancer cases and 10 million cancer deaths worldwide in 2020) [15,16], neurodegenerative disease (an estimated 50 million people worldwide) [17], and vitiligo (affects between 0.5% to 2% of the global population) [18]. TXNDC5 expression is induced under hypoxic conditions in disease states, such as RA, non-small cell lung cancer, and colorectal cancer, and also enhanced expression of TXNDC5 promotes the redox-sensitive activation of cardiac fibroblasts and the augmentation of cardiac fibrosis [1,2,4]. Additionally, TXNDC5 has shown promise in the diagnosis and treatment of the aforementioned diseases by reducing oxidative stress and levels of inflammatory cytokines [2,19].

**Figure 1:**
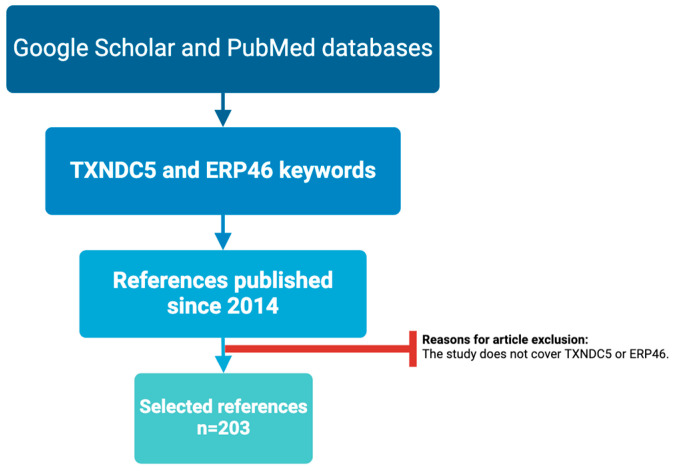
The process of information collection flow chart. Two sources of data were considered: Google Scholar and PubMed. The search criteria used were TXNDC5 and ERP46 keywords, with a time filtration since 2014. This scheme was designed using Microsoft Publisher Document version 2010.

This review summarizes the structural and functional characteristics of TXNDC5’s role and its related molecular pathways in multiple diseases. We conclude this review by highlighting potential areas for future research. Figure 1 displays the references selection process using TXNDC5 and ERP46 as keywords in the last 10 years (a total of 203 publications were used).

## 2. TXNDC5 Gene and Protein Structure

The TXNDC5 gene has been identified in a variety of species, even in the venom glands of the habu serpent [20]. Chimpanzees, mice, and rats have ortholog genes that are similar to those found in *Homo sapiens*. However, most of the research conducted in the past decade has been centered on human and mouse cell lines and tissues [21]. The *TXNDC5* gene’s structural features are highly conserved among vertebrates. Many studies have shown that a large number of SNPs associated with the pathology are located in non-coding regions of DNA, such as introns, 5′-UTR, and 3′-UTR. This fact highlights the importance of these regions in regulating gene expression for *TXNDC5* [21,22]. Based on the genomic sequence assembly, version GRCh38.p14 of the *Homo sapiens* (NCBI, National Center for Bioinformation of the United States), the *TXNDC5* gene spans on chromosome 6p24.3 with a length of 29,272 bp, contains 10 exons and 9 introns and encodes six transcripts, two of which, TXNDC5-isoform 1 and TXNDC5-isoform 3, are translated into proteins. TXNDC5-isoform 1 has 432 amino acids, while TXNDC5-isoform 3 has 324 amino acids [2,21]. Based on the genomic sequence of the *Mus musculus* (version GRCm39 of strain C57BL/6J, NCBI), the *Txndc5* gene located on chromosome 13 with a length of 28,559 bp contains 10 exons and 9 introns and encodes TXNDC5-isoform 1, TXNDC5-isoform 2 and TXNDC5-isoform 3, that can be translated into 417, 323 and 344 amino acid proteins, respectively [21]. A human 6p25.1p24.3 microdeletion associated with mild mental retardation, facial dysmorphism, hypopigmentation of the abdominal skin, cardiac defects, mild pontine hypoplasia and hypotonia, involves loss of *TXNDC5* and 13 other genes OMIM (Table 1) [23]. According to the NCBI database, the *BLOC1S5* gene and its downstream gene *TXNDC5* on the minus strand of the human 6p24.3 region produce a *BLOC1S5-TXNDC5* RNA, while the *BLOC1S5* and its upstream gene *EEF1E1* produce an *EEF1E1-BLOC1S5* RNA. However, no RNA that contains sequences of all three genes, i.e., *EEF1E1-BLOC1S5-TXNDC5* RNA, has been reported to date [24].

**Figure 2:**
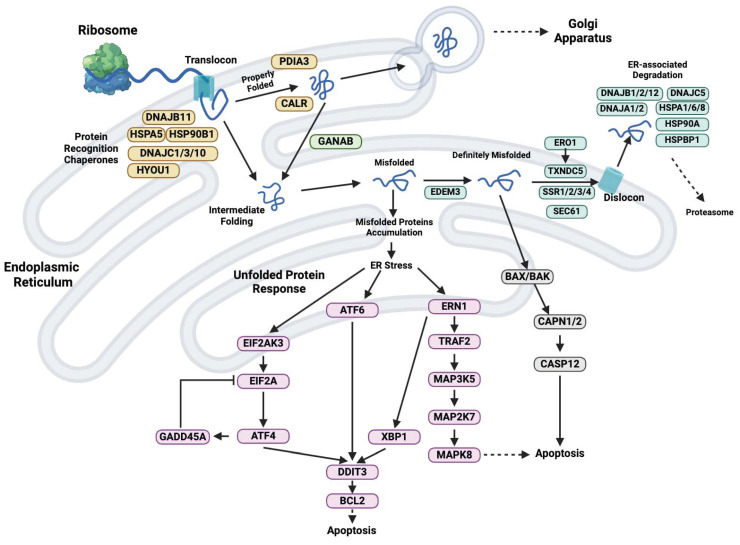
Endoplasmic reticulum protein processing and the action mechanism of TXNDC5. Created with BioRender.com, accessed on 17 February 2024.

Proteostasis refers to the regulation of a balanced and functional proteome through competing and integrated biological pathways within cells. These pathways control the biogenesis, folding, trafficking, and degradation of proteins both inside and outside the cell. ER is a subcellular organelle where proteins are folded with the assistance of lumenal chaperones. Newly synthesized peptides enter the ER through the pore and undergo glycosylation. Proteins that have been folded correctly are packaged into transport vesicles, which then transport them to the Golgi complex (Figure 2) [1,2]. TXNDC5 is a 48 kDa protein primarily expressed in the ER, but it can also be found in lysosomes, vacuoles, cytosol, golgi, mitochondria, and the plasma membrane [1]. Additionally, it can be exported to the extracellular medium. As a protein, TXNDC5 is susceptible to post-translational modifications and catalyzes the rearrangement of disulfide bonds through its disulfide isomerase activity. It reduces the formation of incorrect disulfide bonds in newly folded proteins and catalyzes the oxidation of residues to arrange disulfide bonds in the native structure. This helps proteins fold correctly and protects cells from entering apoptotic pathways [21,25]. In addition, TXNDC5 has chaperone activity that is independent of its isomerase activity. This activity contributes to the oxidative folding of newly synthesized membrane proteins in the ER, providing further support for the antioxidative function of TXNDC5. Additionally, TXNDC5 promotes correct protein folding by participating in electron transfer to other oxidoreductases during oxidation [21,25]. However, it is important to note the role of thioredoxins in bacteria [26] and how their morphological changes could trigger redox alterations inside the cell that could eventually lead to disease [27]. TXNDC5 contains three Trx-like domains (a^0^, a and a′) that all have redox active sites including the CGHC [2,28,29], CxxC and CxxU motifs [30,31,32]. The protein folding process can be accelerated by the three Trx-like domains of TXNDC5, which are connected by approximately 20 amino acid residues that can act independently [2]. Crystallographic and small-angle X-ray scattering studies have revealed that the three Trx-like domains of TXNDC5 are linked by unusually long loops. These loops do not undergo significant interdomain interactions and are arranged in an extended manner to form an open V-shape [3]. The Trx-like domain arrangement present in TXNDC5 has not been found in other PDIs with known structures, suggesting a unique molecular architecture [2,3]. Additionally, the three Trx-like domains appear to function independently in intact TXNDC5, as no cooperative actions were observed [3]. The redox-active sites of TXNDC5 are located separately on the molecular surface and introduce disulfide bonds to unfolded substrates more rapidly and promiscuously than other PDIs [3,28,29,33]. The three catalytic domains of TXNDC5 can bind peptides containing aromatic or alkaline residues. Additionally, the cysteine sites of Trx-like domains Cys88, 217, and 350 are believed to have a protein binding function. The crystal interaction observed in TXNDC5 suggests that the third catalytic domain may bind to the substrate through small hydrophobic pockets on the surface of the Trx-like domain or by exposing the Trp349 residues [2]. TXNDC5 has a specific structural feature, namely the presence of a lysine residue (Lys344) near the second cysteine in the CxxC motif. Furthermore, it was found that the reactivity of Trx-like proteins is not only determined by their CxxC motif. In the third Trx-like domain of TXNDC5, Arg415 forms hydrogen bonds with the carbonyl oxygen of Pro397 instead of inserting into the hydrophobic nucleus. This prevents it from regulating the cysteine pKa value, which is spatially close to the CxxC motif and determines reactivity [2].

## 3. LncRNAs, Circular RNAs and MicroRNAs Targeting TXNDC5

Long non-coding RNAs (LncRNAs) are noncoding transcripts longer than 200 nucleotides that lack protein-encoding ability. Accumulating evidence indicates that LncRNAs have important molecular functions, and that abnormally expressed LncRNAs are related to the metastasis, proliferation, apoptosis, and gene transcription of various cancer cells [34]. At the gene expression level, LncRNA-ENST00000556926 showed a positive correlation with TXNDC5. As a result, the expression of TXNDC5 decreased upon the knockdown of LncRNA-ENST00000556926 in the lung [34]. Circular RNAs (circRNAs) are a type of non-coding RNA that function as competing endogenous RNAs. They have a unique continuous covalent closed-loop structure and lack terminal 5′ caps and 3′ tails. In recent years, circRNAs have been extensively studied in tumors [35]. MicroRNAs (miRNAs) are 22-nucleotide non-coding RNAs that negatively regulate target genes by interacting with the 3′untranslated region (3′UTR). They have been shown to negatively regulate circRNAs, and their interaction may be involved in the growth process of various cancer cells [35]. CircRNA_0078710 and circRNA_0000517 promote the development of hepatocellular carcinoma (HCC) by upregulating TXNDC5 via miRNA-431-5p and miRNA-1296-5p, respectively [2,35,36]. Additionally, circRNA-104718 could alleviate the suppression of miRNA-218-5p’s target, TXNDC5, thereby inhibiting the functions of miRNA in HCC progression [37]. Furthermore, in HCC with poor prognosis, BLOC1S5-TXNDC5 acts as endogenous RNAs for miRNA-30c-5p and miRNA-30e-5p to maintain the high expression of TXNDC5 [38]. MiRNA-573 is another miRNA that can affect signaling pathways by downregulating TXNDC5 in the synovial tissue and fluids of rheumatoid arthritis (RA) patients [2,39,40]. Additionally, the down-expression of miRNA-124 is predicted to increase the mRNA level of TXNDC5 in desmoplastic cerebellar medulloblastoma [41], whereas TXNDC5 is increased by long-term androgen-deprivation treatment (ADT)-induced hypoxia through an miRNA-200b-dependent manner in prostate cancer [2,42], and miRNA-92a in atherosclerosis [43]. MicroRNA offset RNAs (moRNAs) are 20-nt-long RNAs derived from sequences located immediately adjacent to miRNAs in the primary miRNAs and are identified by sequencing small RNA. While miRNAs play a critical role in regulating gene expression, moRNAs are currently considered to be a byproduct of miRNAs biogenesis with no known function. However, recent studies suggest that moRNAs could potentially regulate endogenous target mRNAs in mouse and human tissues, as well as in several viruses [44]. In mouse vascular smooth muscle cells (VSMC) and breast cancer cells (MCF-7), the protein abundance of TXNDC5 was significantly decreased by moRNA-21 but not miRNA-21, indicating that some genes are specifically regulated only by miRNA or moRNA-21 [44] (Table 2).

## 4. TXNDC5 and Cancers

### 4.1. TXNDC5 and Prostate Cancer

Prostate cancer (PCa) is the second most common cancer in men worldwide, and its incidence has been steadily increasing. The overexpression of TXNDC5 can promote the growth of castration-resistant prostate cancer (CRPC) [42,45]. Furthermore, TXNDC5 directly interacts with the androgen receptor (AR) protein to increase its stability and enhance its transcriptional activity, thus stimulating the growth of CRPC [42,46]. TXDNC5-mediated CRPC growth can be fully eliminated by AR inhibition, suggesting TXDNC5 upregulation as an escape pathway for aberrant AR reactivation and CRPC growth in the low androgen milieu. Indeed, TXNDC5 is increased by ADT-induced hypoxia through HIF1A [42]. Duivenvoorden et al. found that when TXNDC5 was stably knocked down using shRNA or overexpressed in prostate carcinoma 22Rv1 cells, tumor growth and proliferation in vitro and in BALB/c nude mice were inhibited and accelerated, respectively [47,48]. Additionally, the downregulation and upregulation of TXNDC5 in the corresponding xenografts showed several candidate genes, including *NAAA*, *SH3BP4,* and *ID1*, that were reciprocally up- and downregulated [49]. On the other hand, the overexpression of *TXNDC5* resulted in reduced sensitivity to ER stress when exposed to tunicamycin and thapsigargin. However, TXNDC5 knockout human prostate adenocarcinoma 22Rv1 cells lost the ability to upregulate protein disulfide isomerase after tunicamycin-induced ER stress [48]. Notably, during simvastatin-induced cell death through c-Jun N-terminal kinase (JNK) activation in DU145 human prostate cancer cells, TXNDC5 exhibited an opposite effect to vimentin, RAB1B, HMGCR, HNRNPK, NDRG1, and IDI1, as it was downregulated despite an increase in its molecular weight, suggesting significant post-translational modification [50]. Therefore, detecting TXNDC5 is crucial for early warning of PCa. For this reason, a novel, highly sensitive photoelectrochemical aptsensor was developed for the detection of TXNDC5 by using the nanohybrids (TiO_2_ NRs/NCQDs) of nitrogen-doped carbon quantum dots (NCQDs) and TiO_2_ nanorods as the photo-to-electron conversion medium, and provides a potential diagnostic application for TXNDC5 [45]. Table 3 summarizes TXNDC5 and its related genes in prostate cancer.

### 4.2. TXNDC5 and Breast Cancer

Breast cancer (BC) is currently the most commonly diagnosed cancer worldwide, with increasing incidence and mortality rates [51]. BLOC1S5-TXNDC5, also known as MUTED-TXNDC5, is an example of a conjoined gene (CG) that, accompanied by ALB, ANGPTL7, IL6, and NGB, is one of five prognosis-associated ferroptosis-related genes, a form of iron-dependent regulated cell death caused by the accumulation of lipid-based reactive oxygen species (Table 3). This CG is closely associated with cancer and can serve as a molecular marker for clinical diagnosis and therapeutic targets. In fact, when compared to adjacent non-tumor breast tissues, the expression level of BLOC1S5-TXNDC5 was significantly higher in BC tissue samples, while the expression levels of the other four genes were significantly lower [51]. In BC, NR4A1 acts as a pro-oncogenic factor regulating ITGB1 and TXNDC5; however, methylene-substituted bis-indole derivative 1,1-bis(3′-indolyl)-1-(p-hydroxyphenyl) methane (DIM-C-pPhOH) acts as an NR4A1 antagonist. Using this antagonist, ITGB1 and TXNDC5 proteins were downregulated, and similar results were observed for the induction of NR4A1-responsive genes *CDKN1A, SERPINB5* and *GADD45α* in breast cancer MDA-MB-231 and SKBR3 cells. These findings may help to understand the tumorigenic activities of TXNDC5 in breast tumors [52].

### 4.3. TXNDC5 and Endometrial and Ovarian Cancer

Endometrial cancer is the fourth most common cancer among women and is increasing in most countries [53]. Identifying patients who will respond positively to therapy is a critical step in the treatment of endometrial and ovarian cancer [53,54]. In endometrial cancer cells where NR4A1 is silenced or treated with an NR4A1 antagonist, the major source of reactive oxygen species (ROS) is associated with the downregulation of TXNDC5 and IDH1 and this is supported by a significant increase in ROS and oxidative/ER stress after the silencing of TXNDC5 [55] (Table 3). A direct linkage between TXNDC5 and the inhibition of mTOR has been observed since knockdown of TXNDC5 induced SESN2 and PRKAA1 and decreased the phosphorylation of mTOR 70S6K and S6RP. Therefore, the inhibition of mTOR signaling in endometrial cancer by NR4A1 antagonists is largely attributed to the downregulation of TXNDC5 and the consequent induction of ROS [53,55]. Auranofin (AF) as an anti-inflammatory and anti-cancer drug, upregulated TXNDC5 expression and protein oxidation in ovarian cancer cells where TXNDC5 upregulation compensated for the deficiency of PDI activity in the ER [30,56]. Furthermore, suppression of TXNDC5 increased the sensitivity to carboplatin as a chemotherapy medication, highlighting its potential importance in carboplatin-resistant ovarian cancer cell lines [54].

### 4.4. TXNDC5 and Skin Cancer

Skin aging is caused by both intrinsic and extrinsic factors and can lead to various types of skin cancer. The expression of TXNDC5, Moesin, RhoGDI, and RSU1 decreases with age, while that of Vimentin and FABP5 increases [57]. Cutaneous squamous cell carcinoma (cSCC), actinic keratosis (AK), and Bowen’s disease (BD) are types of non-melanoma skin cancer (NMSC) that result from the uncontrolled growth of abnormal squamous keratinocytes in the epidermis layer of the skin. TXNDC5, accompanied by APOA1, ALB, SERPINA1, HLA-B, and HP, was found to be significantly upregulated in cSCC compared to AK samples, while BD lesions showed moderate to strong cytoplasmic TXNDC5; in contrast, no TXNDC5 was observed in the cytoplasm of normal epidermal cells (Table 3). Therefore, TXNDC5 could be a valuable biomarker for distinguishing between the two types of lesions [58]. On the other side, melanoma and cutaneous T cell lymphomas (CTCLs) represent the most invasive skin cancer type and comprise the most skin-cancer-related deaths [59,60]. TXNDC5 is downregulated by decreasing N6-methyladenosine (m6A) levels as a post-transcriptional modification in RNA after METTL3 knockdown, ultimately inhibiting melanoma progression. This suggests that TXNDC5 may be the downstream target of METTL3-m6A in melanoma and may play an important role in melanoma carcinogenesis [59]. Resistant CTCLs show high levels of histone acetylation, which correlate with the increased expression of TXNDC5. To treat CTCLs, histone deacetylase inhibitors may contribute to upregulating the expression of TXNDC5, STAT4, TNFRSF17, TNFAIP3, GSTM1, and GSTM3 [60]. These findings may have implications for the development of promising therapeutic strategies for the management of patients with skin cancer.

### 4.5. TXNDC5 and Brain Cancer

Aberrant overexpression of TXNDC5 plays an important role in brain tissue and cancer progression [31]. Data mining revealed that the expression of TXNDC5 and multiple genes listed in Table 3 were significantly upregulated in brain cancers compared to normal tissue [61,62]. Gliomas are the most common primary tumors of the central nervous system. The overexpression of TXNDC5 in glioma pathogenesis patterns differs between tumors and healthy tissues and is associated with more aggressive clinical and molecular features, as well as poor therapy [63]. TXNDC5 levels may serve as a predictor of clinical outcome and therapy success and could be used for targeted therapy [63]. In this regard, the effects of graphene oxide on lipid homeostasis in a 3D brain organoid model and the impact of hydroxytyrosol as an antioxidant on the hippocampus of fetuses whose mothers had been supplemented were investigated and the TXNDC5 was significantly downregulated and upregulated in the GO and hydroxytyrosol groups, respectively [64,65]. Other studies have reported that NR4A1 binds to the promoter of TXNDC5; additionally, elevated levels of TXNDC5 mRNA in PRDX4 ablation, which is a specific redox partner, lead to aberrant ER homeostasis in neuronal cells and ultimately impair spatial memory formation [41,66].

### 4.6. TXNDC5 and Colorectal Cancer

Colorectal cancer (CRC) is the third most common type of cancer and occurs when mutations accumulate in critical oncogenes and tumor suppressor genes. TXNDC5 is overexpressed in CRC tissues and therefore considered an oncogene, and this overexpression may be associated with a significant increase in cell proliferation and colony formation and a decrease in apoptosis [67,68]. Hypoxia can induce the formation of ROS, which may induce TXNDC5 expression by upregulating HIF1A; this effect correlates with higher ECM production and promotes CRC cell proliferation and survival under hypoxic conditions, likely by inhibiting the hypoxia-induced ROS/ER stress signaling pathway [67,68,69]. Furthermore, the knockdown of *TXNDC5* markedly increased hypoxia-induced ROS generation, and the expression of hypoxia-induced ER markers such as DDIT3, HSPA5, ATF4, BAX, and CASP8 [67]. Piperlongumine and DIM-C-pPhOH are anticancer agents or specifically NR4A1 antagonists that act primarily as inducers of ROS by downregulating the pro-reductant IDH1, TXNDC5, and SESN2 which activate the AMPK and inhibit mTOR activation in CRC [70,71]. Additional research indicates that PRDX4 selectively oxidizes TXNDC5 in colitis [72]. Moreover, the co-expression of EDEM3 and TXNDC5 seems to promote the creation of disulfide bonds in the mannosidase homology domain of EDEM3, which is necessary for EDEM3 to display α1,2-mannosidase activity [32,73]. These findings suggested that TXNDC5 functions as an important stress survival factor to maintain the tumorigenesis of CRC cells under hypoxia by regulating hypoxia-induced ROS/ER stress signaling [67]. Table 3 summarizes TXNDC5 and its related genes in colorectal cancer.

### 4.7. TXNDC5 and Cervical Cancer

Cervical cancer (CC) is a prevalent gynecological cancer worldwide and a leading cause of cancer-related deaths in developing countries [74,75]. A significant association was found between the rs408014 and rs7771314 SNPs at the TXNDC5 locus and cervical tumor susceptibility. The overexpression of this gene stimulates cell migration, vasculogenic mimicry, angiogenesis, and reduces apoptosis by downregulating SERPINF1 and TRAF1 expression in CC [76,77]. Additionally, METTL3 enhances the progression of CC by inhibiting ER stress-induced apoptosis and autophagy through the regulation of m6A modification and the upregulation of TXNDC5 and EDEM3 activity [73,74]. Most studies in CC suggest that PRDX4 metabolizes H_2_O_2_ and oxidizes PDI family enzymes upon returning to the reduced state, and it partners with TXNDC5 [78,79,80]. The intermolecular disulfide bond linking PRDX4 is reduced, and the reaction is accelerated by TXNDC5 when the complex is translocated to the ER [79]. In contrast, GPx7 significantly oxidizes TXNDC5 even in the presence of other PDIs [80,81,82]. On the other hand, ERO1α oxidizes TXNDC5 to a lesser extent, while PRDX4’s preferred oxidation substrate is TXNDC5 [78,83,84]. Mutations of Val101 or Trp272, as well as mutations of Cys208 and Cys241, resulted in the inability of ERO1α to oxidize and affect the thermodynamic stability and overall shape of ERO1α, respectively [75,78]. Accordingly, the mutation of Val101 significantly reduces ERO1α-TXNDC5 complexes in CC, while the mutation of Trp272 causes fewer changes in complex formation [75]. Additionally, the Cys208-Cys241 disulfide is preferentially reduced by TXNDC5 [78]. In particular, the deletion of the oxidases TXNDC5 and ERO1α through an electron cascade between proteins in which TXNDC5 takes electrons from a substrate and transfers them to an ERO1α hub complex profoundly suppresses the activity of ITPR1. The ITPR1 also known as a IP3R1 plays a crucial role in Ca^2+^ signaling and its regulation is fundamental for defining the appropriate Ca^2+^ release. Moreover, TXNDC5 and DNAJC10 were identified as an activator of ITPR1, and the direct negative regulator of ITPR1, respectively [85]. Another study found that tris(1,3-dichloro-2-propyl)phosphate (TDCPP), an organophosphate commonly used as a flame retardant which is semi-volatile and continuously released from products, potentially contributes to the development of various diseases, such as CC by the upregulation of LIF and downregulation of TXNDC5, NDUFA5, RLIM, and SERF1A [77]. These findings may be useful for understanding the TXNDC5 tumorigenic activities in cervical tumors [76]. TXNDC5 and its related genes in cervical cancer are listed in Table 3.

### 4.8. TXNDC5 and Lung Cancer

Lung cancer, the second most common cancer in both men and women, the deadliest form of malignancy, and a major public health concern due to the limited therapeutic options, refs. [86,87] increases due to pulmonary fibrosis (PF) which is a condition characterized by excessive scarring of the lungs [88]. Mortality rates for lung cancer, particularly non-small cell lung cancer (NSCLC), have decreased significantly in recent years due to the development of molecular targeted therapies and immunotherapies [87]. Long-term exposure to crystalline silica (CS) can lead to silicosis, a disease characterized by progressive pulmonary fibrosis. This fibrosis is caused by ER stress-related ERN1-TXNDC5 signaling in fibroblast activation. The protein expression level of TXNDC5 was significantly upregulated in the CS-treated group and lung tumor tissues and suppressed by tauroursodeoxycholic acid, as an ER stress inhibitor [86,88,89,90,91] (Table 3). On the other hand, ERN1-XBP1 signaling was closely related to TXNDC5. Therefore, pharmacological inhibition of ERN1 endoribonuclease activity by 4μ8C, a cleaver of XBP1 mRNA to produce an active transcription factor, in addition to knockdown of XBP1 expression, reduces TXNDC5 expression in activated fibroblasts. This information suggests that ERN1 may regulate TXNDC5 by splicing XBP1 mRNA in PF [89]. A study found that knocking down TXNDC5 along with SRXN1 reduced the time needed for splicing XBP1 and spliced XBP1, resulting in the decreased expression of HSPA5 [91,92], and among the handful of proteins that interact with SRXN1, PRDX6, PDIA6, and TXNDC5 were identified in lung cancer [86,91]. In response to ER stress, SRX exhibits an increased association with TXNDC5, facilitating the retention of SRXN1 in the ER. However, TXNDC5 and PDIA6 proteins participate in oxidative protein folding. It is noteworthy that the knockdown of TXNDC5 sensitizes human lung cancer cells to ER stress-induced cell death, inhibits cell proliferation, and represses anchorage-independent colony formation and migration. Conversely, it increases cell invasion and MAPK activation [86,91]. Furthermore, mechanistic investigations have shown that TXNDC5 promotes fibrogenesis by enhancing TGFβ1 signaling through direct binding with and stabilizing TGFBR1 in lung fibroblasts. Additionally, TGFβ1 stimulation can upregulate TXNDC5 via ER stress/ATF6-dependent transcriptional control in lung fibroblasts, leading to excessive activation, proliferation, and ECM production [88,93]. Finally, TXNDC5 is a potential tumor-specific antigen for developing mRNA vaccines and an approach in designing a multi-epitope vaccine targeting TXNDC5 can potentially contribute to NSCLC [87,94]. These studies suggest that targeting TXNDC5 could be a powerful novel approach to ameliorate pulmonary fibrosis, respiratory dysfunction, and lung cancer treatment [86,88,91].

### 4.9. TXNDC5 and Soft Tissue Cancer

Rhabdomyosarcoma (RMS) is the most common soft tissue cancer observed in children and adolescents, representing over 50% of soft tissue sarcomas in children, and fibrosarcoma may be a contributing factor to it [95,96,97]. NR4A1 regulates the expression of the reductant genes TXNDC5 and IDH1 [95,96]. These genes are downregulated in RMS following NR4A1 knockdown or inhibition. Silencing or downregulation of TXNDC5 and IDH1 also induces ROS accumulation and IL24 expression, which is downstream from TXNDC5/IDH1 in RMS and inhibition of cell growth and migration and induction of apoptosis observed after silencing of TXNDC5/IDH1 is attenuated by silencing IL24 [95]. Another study shows that NR4A1 activates mTOR by binding and inactivating TP53, regulates TXNDC5 and IDH1 genes to decrease oxidative and ER stress through the reduction in several markers of ER stress including EIF2AK3, HSPA5, ATF4, and DDIT3, and regulates the expression of growth-promoting/survival genes such as E2F1, CCND2, BIRC5, CDK4, STAT5A, and EGFR through NR4A1-SP1 interactions [41,96,98]. In contrast, NR4A1 antagonists result in decreased interactions of NR4A1, EP300, and POLR2A with the TXNDC5 and IDH1 promoters and also some loss of SP1 from the TXNDC5 promoter [96]. Finally, in muscle cells, TXNDC5 is likely the conduit between ERO1α and the ER disulfide exchange proteins through extensive interactions with HCP5, TXNDC12, and DNAJC10 [97]. Additionally, *TXNDC5* mRNA is associated with AGO2, which exhibits endoribonuclease activity and can cleave target mRNA [44]. The findings indicate that TXNDC5 plays a role in regulating gene expression in soft tissue cancer, revealing a new level of intricacy in this process. Table 3 indicates TXNDC5 and its related genes in soft tissue cancer.

### 4.10. TXNDC5 and Kidney Cancer

Renal fibrosis is a common pathological manifestation of most types of chronic kidney diseases (CKD), characterized by tubulointerstitial fibrosis and glomerular sclerosis, and also is a risk factor for cancer [99]. Kidney cancer is a complex and heterogeneous disease, with renal cell carcinoma (RCC) being the most common and lethal among the common urological cancers [100,101]. TXNDC5 expression is upregulated in RCC tissues and fibrotic kidneys [102]. Importantly, TXNDC5 expression is negatively correlated with the overall survival of patients and induces cell growth, migration, and invasion of RCC and CKD cells [99,101]. TXNDC5, which is transcriptionally controlled by the ATF6-dependent ER stress pathway, mediates its profibrogenic effects by enhancing TGFβ1 signaling activity through post-translational stabilization and the upregulation of TGFBR1 in kidney fibroblasts. This leads to excessive myofibroblast transdifferentiation, proliferation, and ECM production [99]. Additional studies have shown that the protein TXNDC5, which is dependent on ATF6, creates a positive feedback loop that stimulates the activation of TGFβ1/SMAD3, and loss of TXNDC5 significantly prevents fibrosis progression and helps maintain optimal renal function [103,104]. Moreover, inhibiting or enhancing ATF6 signaling in renal tissue can, respectively, downregulate the expression of TGFBR1 and TXNDC5 through the ATF6/TXNDC5 signaling axis [103,105]. TXNDC5 and several genes (Table 3) are regulated by NR4A1 through mTOR signaling in CKD [41,100]. However, calcineurin inhibitors increase TXNDC5 as an optimal immunosuppression for mTOR [106]. Another study reveals that TXNDC5, NR4A1, HCP5, AMACR, TUG1, P2RX5, and SNHG17 are upregulated in diabetic kidney disease [107]. Several studies have reported that TXNDC5 covalently binds to EDEM3 and activates its mannosidase activity. This activation is dependent on the redox activity of TXNDC5 and involves the formation of a disulfide bond between the cysteine residues of the TXNDC5 redox-active sites and the EDEM3 mannosidase domain in kidney cells [108,109,110]. Several ER proteins, such as TXNDC5, HYOU1, ERO1L, HSPA5, EIF2S1, ATF4, ATF6, and DDIT3 are significantly increased in autosomal dominant tubulointerstitial kidney disease [111,112]. In contrast, the expression of TXNDC5 is reduced in kidney cells infected with a pseudorabies vaccine strain [113]. Additionally, the interaction between TXNDC5 and IL12A, IL23α, AGO2 and ITPR1 has been observed in kidney cells [44,85,114]. In summary, these results suggest that targeting TXNDC5 could be a novel and powerful approach to treating or preventing renal fibrosis and RCC.

### 4.11. TXNDC5 and Liver Cancer

Liver cancer, a major global health problem with the second highest mortality rate among all cancers, is a malignant tumor characterized by high rates of metastasis and recurrence. As a common primary liver cancer, hepatocellular carcinoma (HCC) occurs in response to chronic liver injury caused by viral infection, alcohol use, liver fibrosis (LF), non-alcoholic steatohepatitis and non-alcoholic fatty liver disease (NAFLD) [35,37,38,115,116,117]. The *TXNDC5* (rs1225943) AA genotype was the most common genotype in HCC, and the serum levels of TXNDC5 were significantly higher in patients with HCC [115]. TXNDC5 correlates with hepatic steatosis and redox control in the liver, showing increased liver mass with higher fat content associated with SAA1, SAA2 and APOA1 expression [118]. Furthermore, TXNDC5 could couple with the control of APOB levels through the oxidative stress pathway to exert its effect on subsequent hepatic lipid metabolism [119]. TXNDC5 interacts with PRDX4, PRDX6 and HSPA9 to regulate the glutathione metabolism and lipid peroxidation in liver cells [120,121]. TXNDC5 deficiency decreased PRDX6 and HSPA9 protein levels. In addition, lipid peroxidation, glutathione and iPLA_2_ activities were significantly decreased in TXNDC5-deficient cells [120]. Another study identified HSPA5, TXNDC5 and GSTM3 as potent specific CRELD2 binding partners for hepatic metabolic homeostasis [122]. Increased TXNDC5 promotes liver fibrogenesis by inducing hepatic stellate cell (HSC) activation/proliferation, extracellular matrix protein production, and by rendering activated HSCs resistant to apoptosis through the redox-dependent activation of JNK and STAT3, two fibrogenic molecules downstream of the non-canonical TGFβ pathway. In addition, TGFβ1 stimulation upregulates TXNDC5 in HSCs through increased ER stress levels and ATF6-dependent transcriptional control [116,122,123]. On the other side, TXNDC5 is involved in several therapeutic pathways; for example, squalene effectively protects liver cells against oxidative and endoplasmic reticulum stress in a TXNDC5-dependent manner through ERN1 and EIF2AK3 downregulation, and also CALR and APMAP are positively associated with lipid droplets in the presence of squalene and they are decreased in the absence of TXNDC5 [124,125,126,127]. TXNDC5 is efficiently eliminated by the addition of N-(2-hydroxy-5-methylphenyl)-3-phenylpropanolamine as an ATF6 activator [105]. TXNDC5 is involved in reducing the efficiency of photothermal therapy, a minimally invasive and highly specific antineoplastic treatment, by resisting oxidative stress damage and promoting tumor growth and metastasis [128]. In addition, glucagon-like peptide-1 protects liver cells against NAFLD by significantly upregulating the expression of TXNDC5 and downregulating the ER stress marker [129]. Other studies have shown that TXNDC5 is specifically upregulated by SERPINA1 and SREK1 in alpha1-antitrypsin deficiency liver and HCC, respectively [38,130]. Moreover, TXNDC5 expression is downregulated in a high-fat diet and upregulated in cold liver ischemia, respectively [131,132], and it is involved in hepatitis C virus replication, either by affecting lipid metabolism or by translational control via RNA binding [133]. Based on this information, targeting TXNDC5 may be a powerful new approach against LF and preserving hepatic function in patients with HCC [35,116]. TXNDC5 and its related genes in liver cancer are listed in Table 3.

### 4.12. TXNDC5 and Blood Cancer

Unlike many other blood cancers, there is a diverse array of somatic alterations in multiple myeloma (MM) [134,135]. MM is the second most common hematological cancer, which affects terminally differentiated antibody-secreting B cells, known as plasma cells. Clinical manifestations of myeloma include hypercalcemia, anemia, renal failure, and lytic bone lesions [136,137,138,139,140]. TXNDC5 is overexpressed and plays a role in the apoptotic process and shows oxidoreductase activity within MM [140,141]. Other studies revealed the association between TXNDC5 and identified genes indicated in Table 3 that showed significant overexpression in MM [137,141,142]. Interestingly, in multiple studies, overexpression of the TNFSF13 which is associated with a translocation to the 3′ region of TXNDC5, has been observed [139,143,144]. The gene regions most involved in chromoplexy; as a gross structural event that deregulates multiple genes simultaneously and may help explain rapid changes in clinical behavior, are TXNDC5, MYC, NSD2, MAF, CCND1, FCHSD2, NSMCE2 and MAP3K14 [138,140,143,145]. Recently, it has been shown that TXNDC5, a highly mutation-enriched region, acts as a super-enhancer that dysregulates the expression of MYC and GALM, contributing to tumor progression in MM [146,147,148]. Furthermore, multiple studies found that the greatest impact on gene expression in MM occurred when the MYC locus was adjacent to super-enhancers linked to genes like TXNDC5, BMP6, FAM46C, and FOXO3 [149,150,151]. On the other hand, the increased expression of TXNDC5, a protein involved in protection from oxidative stress, plays a major role in bortezomib resistance in MM [141,152]. Additionally, the top differentially regulated genes exposed to YM155, a survivin inhibitor in MM, include DDIT3, ATF3, TXNDC5, SEC24D, and DHX15 [135,152]. Therefore, comprehending the biology of multiple myeloma (MM) and identifying drug-resistance biomarkers, such as TXNDC5, are crucial research challenges that could aid in the development of personalized treatment [135,137,141].

### 4.13. TXNDC5 and Other Types of Cancer

Gastric cancer is a prevalent malignant tumor globally in which TXNDC5 overexpression contributes to cell invasion and metastasis and may correlate with poor tumor differentiation and prognosis [153,154]. Esophageal squamous cell carcinoma (ESCC) is a highly aggressive and lethal malignancy worldwide that KCNH2 contributes to the poor prognosis of ESCC by promoting ESCC cell proliferation, migration, and invasion via TXNDC5 through PI3K and AKT phosphorylation [155]. However, TXNDC5 overexpression does not significantly stimulate dendritic cell maturation, cytokine-induced killer cell development, or cytotoxicity, which are the major types of cells used in immunotherapy for gastric cancer [156,157]. Also, TXNDC5 and YAP1 high expression in endothelial cells stimulates cell proliferation and angiogenesis [158]. Several studies have shown that treatment with NR4A1 inactivators and the knockdown of NR4A1 and CTNNB1 induced ROS-ER stress-dependent apoptosis through the downregulation of TXNDC5 expression in pancreatic cancer, a devastating disease with many risk factors including pancreatitis, obesity and metabolic syndrome [71,159,160,161,162]. Laryngeal squamous cell carcinoma (LSCC) is a prevalent carcinoma of the head and neck [163]. The findings indicate that ER stress-associated apoptosis is a significant mechanism responsible for the apoptotic effect of cisplatin and cetuximab on LSCC cells. Cetuximab enhances cisplatin-induced ER stress-associated apoptosis through DDIT3 and CASP3 in LSCC cells, mainly by inhibiting the expression of TXNDC5 via gene transcription/promoter level, thereby increasing ROS production [163]. These studies indicate that TXNDC5 could be a promising therapeutic target for various types of cancer.

**Table 3:** TXNDC5 and its related genes in various cancers.

Type of Cancer	Gene Symbols *	Related Pathways	References
Prostate	*HIF1A* ^1^, *NAAA* ^2^, *SH3BP4* ^3^, *ID1* ^4^, *RAB1B* ^5^, *HMGCR* ^6^, *HNRNPK* ^7^, *NDRG1* ^8^, *IDI1* ^9^, *VIM* ^10^	Proteasome-mediated degradation ^1^ Neurotransmitter release cycle ^2^ Cellular responses to stimuli ^3,4^ Cell cycle ^5^ Metabolism of steroids ^6,9^ Processing of capped intron-containing pre-mRNA ^7^ Gene expression ^8^ Cholesterol biosynthesis ^9^ Selective autophagy ^10^	[42,49,50]
Breast	*ALB* ^1^, *ANGPTL7* ^2^, *IL6* ^3^, *NGB* ^4^, *BLOC1S5* ^5^, *NR4A1* ^6^, *ITGB1* ^7^, *CDKN1A* ^8^, *SERPINB5* ^9^, *GADD45α* ^10^	Response to elevated platelet cytosolic Ca^2+ 1^ Apoptotic pathways ^2,3,7,9^ MIF-mediated glucocorticoid regulation ^3^ Intracellular oxygen transport ^4^ Biogenesis of lysosome-related organelles ^5^ Gene expression ^6,10^ Proteasome-mediated degradation ^8^ Selective autophagy ^9^	[51,52]
Endometrial	*NR4A1* ^1^, *IDH1* ^2^, *SESN2* ^3^, *PRKAA1* ^4^	Gene expression ^1,3,4^ Innate immune system ^2^ Cellular responses to stimuli ^3^	[53,55]
Cutaneous squamous cell	*APOA1* ^1^, *ALB* ^2^, *SERPINA1* ^3^, *HLA-B* ^4^, *HP* ^5^, *METTL3* ^6^, *STAT4* ^7^, *TNFRSF17* ^8^, *TNFAIP3* ^9^, *GSTM1* ^10^, *GSTM3* ^11^	Plasma lipoprotein assembly, remodeling, and clearance ^1^ Response to elevated platelet cytosolic Ca^2+ 1,2,3^ Innate immune system ^4,5,7^ Processing of capped intron-containing pre-mRNA ^6^ MIF-mediated glucocorticoid regulation ^8^ Metabolism of proteins ^9^ Glutathione conjugation ^10,11^	[58,59,60]
Brain	*P4HB* ^1^, *PDIA3* ^2^, *PDIA4* ^3^, *PDIA5* ^4^, *PDIA6* ^5^, *ERP27* ^6^, *ERP29* ^7^, *ERP44* ^8^, *TMX1* ^9^, *TMX3* ^10^, *TXNDC12* ^11^, *AGR3* ^12^, *DNAJC10* ^13^, *NR4A1* ^14^, *PRDX4* ^15^	Plasma lipoprotein assembly, remodeling, and clearance ^1^ Antigen processing–cross presentation ^2^ Calnexin/calreticulin cycle ^2^ Translational control ^3,8^ Unfolded protein response ^4,5,6^ Cellular responses to stimuli ^4,5^ Protein disulfide isomerase activity ^7,9,11,13^ Response to elevated platelet cytosolic Ca^2+ 10,12^ Gene expression ^14^ Innate immune system ^15^	[41,61,62,66]
Colorectal	*HIF1A* ^1^, *DDIT3* ^2^, *HSPA5* ^3^, *ATF4* ^4^, *BAX* ^5^, *CASP8* ^6^, *IDH1* ^7^, *NR4A1* ^8^, *PRDX4* ^9^, *EDEM3* ^10^	Proteasome-mediated degradation ^1^ Gene expression ^2^ Unfolded protein response ^2,3^ Response to elevated platelet cytosolic Ca^2+ 3^ Integrated stress response ^4^ MIF-mediated glucocorticoid regulation ^5^ Apoptotic pathways ^6^ Innate immune system ^7,9^ Gene expression ^8^ Calnexin/calreticulin cycle ^10^ Metabolism of proteins ^10^	[32,67,68,69,70,71,72,73]
Cervical	*SERPINF1* ^1^, *TRAF1* ^2^, *METTL3* ^3^, *EDEM3* ^4^, *PRDX4* ^5^, *GPX7* ^6^, *ERO1α* ^7^, *ITPR1* ^8^, *DNAJC10* ^9^, *LIF* ^10^, *NDUFA5* ^11^, *RLIM* ^12^, *SERF1A* ^13^	Apoptotic pathways ^1,2^ Processing of capped intron-containing pre-mRNA ^3^ Chromatin regulation ^3^ Calnexin/calreticulin cycle ^4^ Metabolism of proteins ^4^ Innate immune system ^5,12^ Cellular responses to stimuli ^6,7^ Development angiotensin activation ^8^ Protein disulfide isomerase activity ^9^ MIF-mediated glucocorticoid regulation ^10^ Respiratory electron transport ^11^ Protein aggregation ^13^	[73,74,76,77,78,79,80,81,82,83,84]
Non-small cell lung	*ERN1* ^1^, *XBP1* ^2^, *HSPA5* ^3^, *SRXN1* ^4^, *PRDX6* ^5^, *PDIA6* ^6^, *TGFβ1* ^7^, *TGFBR1* ^8^, *ATF6* ^9^	Cellular responses to stimuli ^1,2,4,5,6,9^ Unfolded protein response ^2,3,6,9^ Response to elevated platelet cytosolic Ca^2+ 3^ Innate immune system ^5^ Apoptotic pathways ^7,8^	[86,88,89,90,91,93]
Rhabdomyosarcoma	*NR4A1* ^1^, *IDH1* ^2^, *IL24* ^3^, *TP53* ^4^, *EIF2AK3* ^5^, *HSPA5* ^6^, *ATF4* ^7^, *DDIT3* ^8^, *E2F1* ^9^, *CCND2* ^10^, *BIRC5* ^11^, *CDK4* ^12^, *STAT5A* ^13^, *EGFR* ^14^, *SP1* ^15^, *EP300* ^16^, *POLR2A* ^17^, *ERO1* ^18^, *HCP5* ^19^, *TXNDC12* ^20^, *DNAJC10* ^21^, *AGO2* ^22^	Gene expression ^1,8,11^ Innate immune system ^2,19^ MIF-mediated glucocorticoid regulation ^3^ Proteasome-mediated degradation ^4^ Cellular responses to stimuli ^5,18^ Unfolded protein response ^6,8^ Response to elevated platelet cytosolic Ca^2+ 6,20^ Integrated stress response ^7^ Apoptotic pathways ^9,14^ Protein kinase binding ^10^ Cellular response to stress ^12^ Prolactin signaling ^13,14,15^ RNA polymerase and promoter opening ^16,17^ Protein disulfide isomerase activity ^20,21^ Transcriptional regulation ^22^ Cell junction organization ^22^	[41,44,95,96,97,98]
Renal	*NR4A1* ^1^, *IDH1* ^2^, *E2F1* ^3^, *CCND2* ^4^, *BIRC5* ^5^, *CDK4* ^6^, *STAT5A* ^7^, *SP1* ^8^, *AGO2* ^9^, *ATF6* ^10^, *TGFβ1* ^11^, *TGFBR1* ^12^, *EDEM3* ^13^, *ITPR1* ^14^, *SMAD3* ^15^, *IL12A* ^16^, *IL23α* ^17^	Gene expression ^1,5^ Innate immune system ^2^ Apoptotic pathways ^3,11,12^ Protein kinase binding ^4^ Cellular response to stress ^6^ Prolactin signaling ^7,8^ Transcriptional regulation ^9^ Cell junction organization ^9^ Cellular responses to stimuli ^10^ Unfolded protein response ^10^ Calnexin/calreticulin cycle ^13^ Metabolism of proteins ^13^ Development angiotensin activation ^14^ Proteasome-mediated degradation ^15^ Mediated glucocorticoid regulation ^16,17^	[41,43,85,99,100,103,104,105,108,109,110,111,112,114]
Liver	*SAA1* ^1^, *SAA2* ^2^, *APOA1* ^3^, *APOB* ^4^, *PRDX4* ^5^, *PRDX6* ^6^, *HSPA9* ^7^, *HSPA5* ^8^, *GSTM3* ^9^, *CRELD2* ^10^, *STAT3* ^11^, *TGFβ1* ^12^, *ATF6* ^13^, *ERN1* ^14^, *EIF2AK3* ^15^, *CALR* ^16^, *APMAP* ^17^, *SERPINA1* ^18^, *SREK1* ^19^	Cholesterol homeostasis ^1,2^ Plasma lipoprotein assembly, remodeling, and clearance ^3,4^ Response to elevated platelet cytosolic Ca^2+ 3,8,10,18^ Innate immune system ^4,5^ Cellular responses to stimuli ^5,7,13,14,15^ Unfolded protein response ^8,10,13,16^ Glutathione conjugation ^9^ Prolactin signaling ^11^ Apoptotic pathways ^12^ Adipocyte differentiation ^17^ Transport to the Golgi and subsequent modification ^18^ Processing of capped intron-containing pre-mRNA ^19^	[38,105,118,119,120,121,122,123,124,125,126,127,129]
Myeloma	*BCL2L11* ^1^, *CCND1* ^2^, *CCND2* ^3^, *LTBR* ^4^, *PDIA3P1* ^5^, *HSP90B1* ^6^, *MZB1* ^7^, *ACTB* ^8^, *P4HB* ^9^, *DERL3* ^10^, *HERPUD1* ^11^, *PDIA4* ^12^, *PDIA6* ^13^, *RRBP1* ^14^, *SSR3* ^15^, *SSR4* ^16^, *UBE2J1* ^17^, *TNFSF13* ^18^, *MYC* ^19^, *NSD2* ^20^, *MAF* ^21^, *FCHSD2* ^22^, *NSMCE2* ^23^, *MAP3K14* ^24^, *GALM* ^25^, *BMP6* ^26^, *FOXO3* ^27^, *DDIT3* ^28^, *ATF3* ^29^, *SEC24D* ^30^, *DHX15* ^31^	Apoptotic pathways ^1,4,7,26^ Proteasome-mediated degradation ^2,10^ Protein kinase binding ^3,27^ Protein disulfide isomerase activity ^5^ Unfolded protein response ^6,13,28^ Cellular responses to stimuli ^6,11,13,29^ Regulation of actin dynamics for phagocytic cup formation ^8^ Plasma lipoprotein assembly, remodeling, and clearance ^9^ PERK regulates gene expression ^11,29^ Translational control ^12^ Signaling receptor activity ^14^ Metabolism of proteins ^15,16,23,24^ Regulation of degradation ^17^ MIF-mediated glucocorticoid regulation ^18,26^ Prolactin signaling ^19^ Homology directed repair ^20^ DNA double strand break response ^20^ Gene expression ^21,28^ Endocytosis ^22^ Galactose metabolism ^25^ Vesicle trafficking ^30^ Processing of capped intron-containing pre-mRNA ^31^	[137,139,140,141,142,143,144,145,146,147,148,149,150,151]
Esophageal squamous cell	*KCNH2*	Potassium channels	[155]
Endothelial cell	*YAP1*	Gene expression	[158]
Pancreas	*NR4A1* ^1^, *CTNNB1* ^2^	Gene expression ^1,2^ Phosphorylation ^2^	[71,159,160,161,162]
Head and neck	*DDIT3* ^1^, *CASP3* ^2^	Gene expression ^1^ Unfolded protein response ^1^ Apoptotic pathways ^2^	[163]

* The gene symbols are followed by superscript numbers that indicate the related gene pathways and some genes may appear in more than one pathway. Abbreviations: HIF1A, hypoxia inducible factor 1 subunit alpha; NAAA, N-acylethanolamine acid amidase; SH3BP4, Sh3 domain binding protein 4; ID1, inhibitor of DNA binding 1; RAB1B, Ras-related protein rab-1b; HMGCR, 3-hydroxy-3-methylglutaryl-coa reductase; HNRNPK, heterogeneous nuclear ribonucleoprotein k; NDRG1, N-myc downstream regulated 1; IDI1, isopentenyl-diphosphate delta isomerase 1; VIM, vimentin; ALB, albumin; ANGPTL7, angiopoietin-like 7; IL6, interleukin 6; NGB, neuroglobin; BLOC1S5, biogenesis of lysosomal organelles complex 1 subunit 5; NR4A1, nuclear receptor subfamily 4 group a member 1; ITGB1, integrin subunit beta 1; CDKN1A, cyclin-dependent kinase inhibitor 1a; SERPINB5, Serpin family b member 5; GADD45α, growth arrest and DNA damage inducible alpha; IDH1, isocitrate dehydrogenase (NADP(+)) 1; SESN2, sestrin 2; PRKAA1, protein kinase amp-activated catalytic subunit alpha 1; APOA1, apolipoprotein a1; SERPINA1, serpin family A member 1; HLA-B, major histocompatibility complex class I b; HP, haptoglobin; METTL3, methyltransferase 3, n6-adenosine-methyltransferase complex catalytic subunit; STAT4, signal transducer and activator of transcription 4; TNFRSF17, Tnf receptor superfamily member 17; TNFAIP3, Tnf alpha induced protein 3; GSTM1, glutathione S-transferase mu 1; GSTM3, glutathione S-transferase mu 3; P4HB, prolyl 4-hydroxylase subunit beta; PDIA3, protein disulfide isomerase family A member 3; PDIA4, protein disulfide isomerase family a member 4; PDIA5, protein disulfide isomerase family a member 5; PDIA6, protein disulfide isomerase family a member 6; ERP27, endoplasmic reticulum protein 27; ERP29, endoplasmic reticulum protein 29; ERP44, endoplasmic reticulum protein 44; TMX1, thioredoxin-related transmembrane protein 1; TMX3, thioredoxin-related transmembrane protein 3; TXNDC12, thioredoxin domain containing 12; AGR3, anterior gradient 3, protein disulfide isomerase family member; DNAJC10, DnaJ heat shock protein family (Hsp40) member c10; PRDX4, peroxiredoxin 4; DDIT3, DNA damage inducible transcript 3; HSPA5, heat shock protein family a (Hsp70) member 5; ATF4, activating transcription factor 4; BAX, Bcl2 associated x apoptosis regulator; CASP8, caspase 8; EDEM3, Er degradation enhancing alpha-mannosidase-like protein 3; SERPINF1, serpin family f member 1; TRAF1; Tnf receptor associated factor 1, METTL3; methyltransferase 3, n6-adenosine-methyltransferase complex catalytic subunit; GPX7, glutathione peroxidase 7; ERO1α, endoplasmic reticulum oxidoreductase 1 alpha; ITPR1, inositol 1,4,5-trisphosphate receptor type 1; LIF, Lif interleukin 6 family cytokine; NDUFA5, NADH: ubiquinone oxidoreductase subunit a5; RLIM, ring finger protein, LIM domain interacting; SERF1A, small edrk-rich factor 1a; ERN1, endoplasmic reticulum to nucleus signaling 1; XBP1, X-box binding protein 1; SRXN1, sulfiredoxin 1; PRDX6, peroxiredoxin 6; TGFβ1, transforming growth factor beta 1; TGFBR1, transforming growth factor beta receptor 1; ATF6, activating transcription factor 6; IL24, interleukin 24; TP53, tumor protein p53; EIF2AK3, eukaryotic translation factor 2 alpha kinase 3; E2F1, E2f transcription factor 1; CCND2, cyclin d2; BIRC5, baculoviral iap repeat containing 5; CDK4, cyclin-dependent kinase 4; STAT5A, signal transducer and activator of transcription 5a; EGFR, epidermal growth factor receptor; SP1, Sp1 transcription factor; EP300, E1a binding protein p300; POLR2A, RNA polymerase II subunit a; HCP5, Hla complex p5; AGO2, Argonaute RISC catalytic component 2; SMAD3, Smad family member 3; IL12A, interleukin 12a; IL23α, interleukin 23 subunit alpha; SAA1, serum amyloid a1; SAA2, serum amyloid a2; APOB, apolipoprotein b; HSPA9, heat shock protein family a (Hsp70) member 9; CRELD2, cysteine rich with egf-like domains 2; STAT3, signal transducer and activator of transcription 3; CALR, calreticulin; APMAP, adipocyte plasma membrane associated protein; SREK1, splicing regulatory glutamic acid and lysine rich protein 1; BCL2L11, Bcl2 like 11; CCND1, cyclin d1; LTBR, lymphotoxin beta receptor; PDIA3P1, protein disulfide isomerase family a member 3; HSP90B1, heat shock protein 90 beta family member 1; MZB1, marginal zone b and b1 cell-specific protein; ACTB, actin beta; P4HB, prolyl 4-hydroxylase subunit beta; DERL3, derlin 3; HERPUD1, homocysteine inducible er protein with ubiquitin-like domain 1; RRBP1, ribosome binding protein 1; SSR3, signal sequence receptor subunit 3; SSR4, signal sequence receptor subunit 4; UBE2J1, ubiquitin conjugating enzyme e2 j1; TNFSF13, Tnf superfamily member 13; MYC, Myc proto-oncogene bhlh transcription factor; NSD2, nuclear receptor binding set domain protein 2; MAF, Maf bzip transcription factor; FCHSD2, Fch and double sh3 domains 2; NSMCE2; Nse2 (MMS21) homolog, smc5-smc6 complex sumo ligase; MAP3K14, mitogen-activated protein kinase 14; GALM, galactose mutarotase; BMP6, bone morphogenetic protein 6; FOXO3, forkhead box o3; ATF3, activating transcription factor 3; SEC24D, Sec24 homolog d copII coat complex component; DHX15, DEAH-box helicase 15; KCNH2, potassium voltage-gated channel subfamily h member 2; YAP1, Yes1 associated transcriptional regulator; CTNNB1, catenin beta 1; CASP3, caspase 3.

## 5. TXNDC5 and Fibrosis-Related Pathologies

TXNDC5 promotes fibrosis in multiple organs, including the heart, lung, kidney, and liver, as an intermediary of TGFβ signaling. TGFβ induces the upregulation of TXNDC5 via increased ER stress levels and ATF6-mediated transcriptional control. This TGFβ/ATF6/TXNDC5 signaling axis highlights the crucial role of TXNDC5 in fibrogenesis. Additionally, the TRX domains of TXNDC5 contribute to the proper folding and stabilization of pro-fibrotic proteins [164]. TXNDC5 expression is upregulated in human cardiac fibroblasts and in mouse hearts with pathological cardiac hypertrophy. TXNDC5 aids in the folding of ECM proteins and activates cardiac fibroblasts through its PDI activity, which is sensitive to redox and activates the JNK signaling pathway. The deletion of TXNDC5 protects against isoproterenol-induced myocardial fibrosis, hypertrophy, and contractile dysfunction in mice. Targeting TXNDC5 expression could potentially inhibit cardiac fibrosis with fewer side effects than targeting TGFβ and RAAS, as TXNDC5 expression is only upregulated in activated cardiac fibroblasts [165]. In addition, TXNDC5 is involved in the progression of pulmonary fibrosis (PF) by modulating TGFβ signaling as well. TGFβ1 stimulation induces TXNDC5 upregulation in lung fibroblasts via increased ER stress levels and ATF6-mediated transcriptional regulation. TXNDC5 enhances the protein stability of TGFBR1 and augments TGFβ signaling, generating a positive feedback loop of the TGFβ1/ATF6/TXNDC5/TGFBR1 signaling axis to cause severe scarring in the lung. The use of the inducible fibroblast-specific deletion of Txndc5 provides additional confirmation of the pathogenic role of fibroblastic TXNDC5 in the development and progression of lung fibrosis [88]. A recent study showed that TXNDC5 regulates the TGFβ/ATF6/TGFβR1 signaling axis in renal fibrosis (RF), similar to what is observed in PF. The ATF6-dependent ER stress pathway transcriptionally regulates TXNDC5 in fibroblasts following TGFβ stimulation. The depletion of TXNDC5 attenuates human kidney fibroblast activation, proliferation, and ECM production induced by TGFβ1. Additionally, targeting TXNDC5 in kidney fibroblasts reduced scarring in multiple RF mouse models [99]. Finally, the role of TXNDC5 in the progression of liver fibrosis (LF) has recently been investigated. TXNDC5 is significantly expressed in activated hepatic stellate cells (HSCs) and at the fibrotic foci of the livers of human patients and mice with liver fibrosis/cirrhosis. TXNDC5 induces HSC activation through ROS-dependent JNK signaling. Additionally, TXNDC5 renders HSCs resistant to apoptosis via STAT3 signaling, leading to the accumulation of activated HSCs and excessive fibrotic scarring in the liver. Inhibiting the catalytic function of TXNDC5 eliminates JNK and STAT3 activation and the subsequent fibrotic responses. Additionally, following acute or chronic liver injury, the pro-fibrotic cytokine TGFβ activates the ER stress pathway. These responses rely on the redox activity of TXNDC5 to initiate TGFβ canonical and non-canonical signaling and stabilize ECM and TGFβR1 proteins, resulting in a positive feedback loop of TGFβ/ATF6/TGFβR1 signaling in LF [116]. These findings suggest that TXNDC5 plays a crucial role in organ fibrosis through four different mechanisms. These mechanisms include facilitating the folding of ECM proteins, stabilizing TGFβR1 protein, inducing fibroblast activation and proliferation through TGFβ non-canonical JNK signaling, and activating phosphorylated STAT3 to make fibroblasts resistant to apoptosis.

## 6. TXNDC5 and Rheumatoid Arthritis

Rheumatoid arthritis (RA) is a chronic inflammatory joint disease that is characterized by joint damage, hyperplasia of synovial tissue, irregular angiogenesis, and bone damage [166,167,168]. TXNDC5 accompanied by multiple genes (table in Section 8) are key players in ER stress associated with synovitis in osteoarthritis, chronic pyrophosphate arthropathy, and rheumatoid arthritis [169,170,171]. TXNDC5 responds to ER stress in RA synovial fibroblast-like cells (RASFs) as its expression is induced by ER stress at both endogenous and secretory levels, whereas, silencing TXNDC5 attenuated the induction of IL6 and CXCL8 from RASFs in response to ER stress [40,167]. TXNDC5 was detected in the exosomes from RASFs and its content in exosomes increased when RASFs were stimulated by ER stress inducers through the increased production of inflammatory factors and the phosphorylated levels of ERK, PKB/Akt, NF-κB, and MAPK signaling pathways [172]. On the other side, TXNDC5 expression in RASFs is significantly upregulated in response to lipopolysaccharides (LPS), TNF-α and IL6 [173,174]. Furthermore, TXNDC5 directly interacts with HSPA8 to sequester it in the cytoplasm, and HSPA8 activates NF-κB signaling by destabilizing IκBβ protein in the absence of LPS or facilitating its nuclear translocation in the presence of LPS in RASFs [174]. Additional studies showed that PCSK6 regulates the expression of HIF1A and TXNDC5, and the downregulation of TXNDC5 could contribute to RASF antiangiogenic and proapoptotic features through the suppression of CXCL10 and TNFSF10 [175,176]. The inhibition of TXNDC5 expression in RASFs leads to an increase in IGFBP1 expression. Therefore, the increased expression of TXNDC5 in the RA synovium in response to hypoxia stimulation downregulates IGFBP1 expression, resulting in increased IGF activity. Consequently, RA exacerbates metabolic disorders such as diabetes mellitus, indicating a close association between RA and diabetes mellitus [166,168].

## 7. TXNDC5 and Diabetes Mellitus

Diabetes mellitus (DM) is a condition characterized by peripheral insulin resistance, hyperglycemia, and defective insulin secretion. Insulin-producing pancreatic β-cells have a highly developed ER and are therefore affected by ER stress under hyperglycemic conditions [177]. TXNDC5 has long been considered a gene that contributes to diabetes susceptibility (table in Section 8). It plays a major role in regulating insulin content and may also contribute to glucose toxicity by affecting insulin production [121,168] so that loss-of-function variation in TXNDC5 elevates fasting blood glucose levels [178]. On the other side, casein as a dietary protein source for type 2 diabetes, can downregulate TXNDC5 [179]. TXNDC5 is closely associated and interacts with proinsulin in pancreatic islets. The reduction in TXNDC5 under high glucose conditions is restored by liraglutide, such as GLP-1. This suggests that ATF6 and XBP1, through consensus motifs of the TXNDC5 promoter that can be recognized by them, can contribute to UPR involved in the differential expression of TXNDC5 under high glucose conditions [177]. TXNDC5 is involved in the disulfide formation of proinsulin as a downstream target of the ERN1–XBP1 pathway. Therefore, ERN1 is required for the increased expression of TXNDC5 in pancreatic β cells [180]. Additionally, TXNDC5 can regulate the activity of IGF1 [168]. These findings suggest a new pathogenetic mechanism of TXNDC5 triggered by glucotoxicity and offer new targets for future therapeutic interventions [177].

## 8. TXNDC5 and Heart Diseases

Heart failure (HF) is a significant and expanding public health issue caused by cardiac fibrosis (CF) and atherosclerosis [165]. Atherosclerosis is a progressive inflammatory disease that causes the accumulation of lipids in the arterial intima and the build-up of atherosclerotic plaques, leading to human morbidity and mortality [181]. Atherosclerotic lesions typically occur in specific arterial regions where disturbed blood flow (DF) activates endothelial cells (ECs) via mechanotransduction, resulting in peripheral artery disease, carotid artery disease, and ischemic stroke [43,182]. TXNDC5 promotes cardiac fibrosis by facilitating ECM protein folding [4]. Additionally, it triggers cardiac fibroblast activation and proliferation through JNK signaling, which is dependent on NOX4-derived ROS and independent of SMAD3 [165]. Cardiac TXNDC5 expression increases in hypertrophic and failing hearts, likely under the control of a TGFβ1/ER stress/ATF6 signaling axis. This contributes to the excessive accumulation of myofibroblasts and ECM proteins that lead to cardiac fibrosis [183]. Various studies have shown that both DF and hyperlipidemia have a significant impact on the endothelial expression of TXNDC5, and also TXNDC5 mediates TNF-α-induced NOX activation in ECs [43,182,184]. However, the TXNDC5 protein level, when exposed to aeroplysinin-1 as a modulator of the redox balance in ECs, is downregulated [185], and a chicory root diet increased it in porcine aorta [186]. TXNDC5 promotes endothelial activation and atherogenesis induced by DF. It enhances the ubiquitination and proteasome-mediated degradation of the transcription factor HSF1, resulting in a decreased expression of HSP90AB1 and the reduced stability of NOS3 and KLF2 proteins [181,182]. Other evidence suggests a role for TXNDC5 in platelet function and arterial thrombosis through enhanced ITGA2B activation by targeting disulfide bonds, platelet aggregation and ATP release [187,188]. The expression of TXNDC5 on the platelet surface increases with thrombin stimulation, while PGHG, as an inhibitor of PDI reductase activity, suppressed TXNDC5 [187,189]. Furthermore, PDIA6-deficient platelets were seen to increase in HSPA5, CALR, and TXNDC5 [190]. Therefore, targeting TXNDC5 could be a powerful new therapeutic approach to mitigate excessive CF and dysfunction, thereby improving cardiac function and outcomes in patients with HF and atherosclerotic cardiovascular diseases [165,181,182]. This information is summarized in Table 4.

**Table 4:** TXNDC5 and its associated genes in rheumatoid arthritis, diabetes mellitus and heart diseases.

Diseases	Gene Symbols *	Related Pathways	References
Rheumatoid arthritis	*DNAJB11* ^1^, *CALR* ^2^, *ERP29* ^3^, *GANAB* ^4^, *HSP90B1* ^5^, *HSPA1A* ^6^, *HSPA5* ^7^, *HYOU1* ^8^, *LMAN1* ^9^, *PDIA4* ^10^, *CXCL8* ^11^, *IL6* ^12^, *TNF-α* ^13^, *HSPA8* ^14^, *PCSK6* ^15^, *HIF1A* ^16^, *CXCL10* ^17^, *TNFSF10* ^18^, *IGFBP1* ^19^	Unfolded protein response ^1,2,5,7,8^ Cellular responses to stimuli ^1,5,8,19^ Processing of secretory proteins ^3^ Translation of structural proteins ^4^ Proteasome-mediated degradation ^6,16^ Response to elevated platelet cytosolic Ca^2+ 7^ Signaling by Rho GTPases ^9^ Translational control ^10^ Apoptotic pathways ^11,12^ MIF-mediated glucocorticoid regulation ^11,12,13,17,18^ Processing of capped intron-containing pre-mRNA ^14^ Selective autophagy ^14^ Plasma lipoprotein assembly, remodeling, and clearance ^15^ Gene expression ^19^	[40,166,167,168,173,174,175,176]
Diabetes mellitus	*GLP-1* ^1^, *ATF6* ^2^, *XBP1* ^3^, *ERN1* ^4^, *IGF1* ^5^	Integration of energy metabolism ^1^ Unfolded protein response ^2,3^ Cellular responses to stimuli ^2,3,4^ Apoptotic pathways ^5^	[168,177,180]
Heart diseases	*ATF6* ^1^, *TNF-α* ^2^, *NOX4* ^3^, *SMAD3* ^4^, *TGFβ1* ^5^, *HSF1* ^6^, *HSP90AB1* ^7^, *NOS3* ^8^, *KLF2* ^9^, *ITGA2B* ^10^, *PDIA6* ^11^, *HSPA5* ^12^, *CALR* ^13^	Unfolded protein response ^1,11,12,13^ Cellular responses to stimuli ^1,3,11^ MIF-mediated glucocorticoid regulation^2^ Proteasome-mediated degradation ^4^ Apoptotic pathways ^5,10^ Cellular response to heat stress ^6^ Selective autophagy ^6^ Inflammasomes ^7^ Metabolism of nitric oxide ^8^ Cell differentiation ^9^ Response to elevated platelet cytosolic Ca^2+ 12^	[43,180,181,182,183,184,185,188,189,191]

* The gene symbols are followed by superscript numbers that indicate the related gene pathways and some genes may appear in more than one pathway. Abbreviations: DNAJB11, DnaJ heat shock protein family (Hsp40) member b11; ERP29, endoplasmic reticulum protein 29; GANAB, glucosidase II alpha subunit; HSP90B1, heat shock protein 90 beta family member 1; HSPA1A, heat shock protein family a (Hsp70) member 1a HYOU1, hypoxia upregulated 1; LMAN1, lectin mannose binding 1; CXCL8, C-X-C motif chemokine ligand 8; TNF-α, tumor necrosis factor alpha; HSPA8, heat shock protein family a (Hsp70) member 8; PCSK, proprotein convertase subtilisin/kexin type 6; CXCL10, C-X-C motif chemokine ligand 10; TNFSF10, Tnf superfamily member 10; IGFBP1, insulin-like growth factor binding protein 1; GLP-1, glucagon-like peptide 1 receptor; IGF1, insulin-like growth factor 1; NOX4, Nadph oxidase 4; HSF1, heat shock transcription factor 1; HSP90AB1, heat shock protein 90 alpha family class b member 1; NOS3, nitric oxide synthase 3; KLF2, Kruppel-like factor 2; ITGA2B, integrin subunit alpha 2b.

## 9. TXNDC5 and Other Disorders

TXNDC5 is also involved in other diseases; for instance, the reduced accumulation of collagen-I in the testis is likely due to the inhibition of TGFβ1, αSMA, and ER protein expression of TXNDC5 [191]. In addition, the mapping of breakpoints in balanced chromosomal translocations through shallow whole-genome sequencing identifies TXNDC5 as a potential gene responsible for human Mendelian disorders with the clinical criteria consisting of developmental delay, neurological dysfunction, congenital anomalies and/or dysmorphic features [192]. Enterovirus 71-infected hand, foot, and mouth upregulates the expression of genes such as TXNDC5, IFI27, CD177, CD27, TMIGD2, and SIT1 [193]. TXNDC5 is downregulated by the upregulation of PRKG1 in the development of adolescent idiopathic scoliosis and is also accompanied by PRDX6, PARK7, cathepsin D, and MVP as part of the common circuit that helps senescent cells survive after stress in the bone marrow [194,195]. TXNDC5 is also decreased in immune reconstitution inflammatory syndrome [196]. Furthermore, PIT1 co-localized with TXNDC5 is required for ER homeostasis, chondrocyte survival, and skeletal development [197], also TXNDC5 knockdown is highly toxic to osteosarcoma cells, possibly implying that it has a critical function in cells that endogenously express collagen-I [198]. Other studies have identified an upregulation of TXNDC5 in Parkinson’s disease, respiratory distress syndrome following cardiopulmonary bypass, COVID-19, malaria, *Salmonella enteritidis* and Marek’s disease infection [199,200,201,202,203,204]. However, TNFRSF17 and TXNDC5 are the target genes for the COVID-19 vaccine [205]. TXNDC5 is upregulated in patients with septic shock compared to septic patients without shock or healthy controls; this suggests that inhibiting TXNDC5 can attenuate lipopolysaccharide-induced septic shock by inhibiting the NF-κB signaling pathway [206]. Additionally, it was found that the TXNDC5 level is significantly higher in patients with both early-onset and late-onset pre-eclampsia pregnancies and systemic lupus erythematosus [207,208]. Furthermore, an SNP associated with the age of menopause, TXNDC5: rs1319689, was identified [209]. Recently, major interactions and upregulation were discovered in HDAC9\10 and fibulin-3 with TXNDC5 in hypothyroidism and retinal degeneration, respectively [210,211,212]. In summary, the presented data suggest potential diagnostic applications for TXNDC5; therefore, targeting TXNDC5 could be a powerful new approach against multiple diseases.

## 10. Conclusions

**Figure 3:**
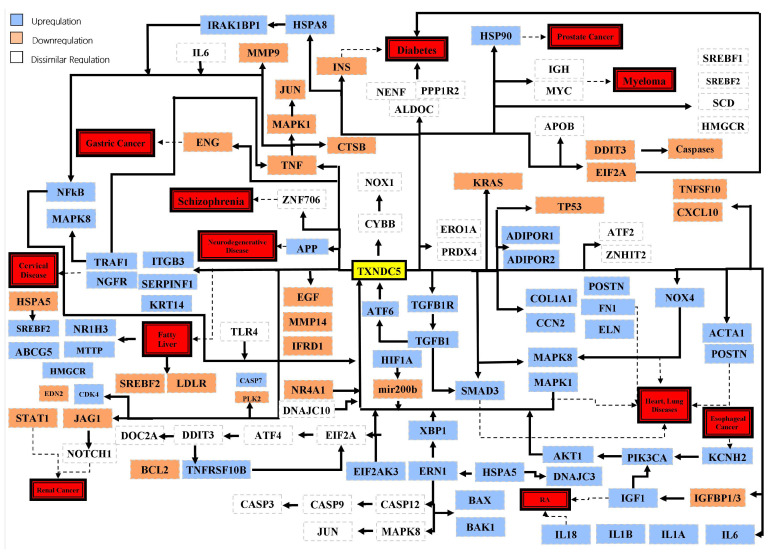
This is a general summary of pathways that point to genes related to TXNDC5, adapted from multiple studies on different disorders. Each arrow indicates the association of one gene with another, and dashed arrows indicate the outcome of each pathway to certain disorders. Blue indicates the upregulation of genes in the overexpression of TXNDC5, while orange indicates downregulation. The white color indicates that the regulation of those genes differs among organs, as shown by the studies considered. This schematic figure was created using Microsoft Publisher Document Version 2010 [21,36,40,42,50,67,76,86,88,89,99,101,155,163,165,166,172,174,176,180,213,214,215,216].

Transcriptional regulation pathways are complex mechanisms that control gene expression and play important roles in various physiological processes and pathologies. The roles of TXNDC5 in these pathways are gaining attention due to their implications in a range of disorders, from metabolic syndromes to neurodegenerative diseases. To summarize, Figure 3 displays the pathways pointing to TXNDC5 roles by adapting the multiple studies in different disorders presented in this review. The research carried out in the last few years has shown that TXNDC5 contributes to a tumor cell phenotype that can adapt to cellular stress. This responsiveness to cellular stress cues may explain why TXNDC5 levels vary in specific cancers. It is unclear how the overall metabolism of tumor cells is connected to chemoresistance and the process of metastasis. However, TXNDC5 can play a role in these processes due to its capacity for rapid responses and involvement in numerous downstream pathways. We believe that the various coactivation partners—in addition to NR4A1, EDEM3, IRE1, XBP1, ATF6, MTTL3, ERO1, IDH1 and TGFβ1—in the upstream and downstream gene expression networks of TXNDC5 need to be examined to better understand the fine-tuned, and possibly tissue- and/or clone-dependent, specifics of TXNDC5-mediated effects. The oxidative and ER stress metabolisms are particularly often found to be downregulated in tumor cells with a high expression of TXNDC5; in consequence, TXNDC5 protects tumor cells against apoptosis. Finally, it has been concluded that in cancer, TXNDC5 acts as a foe, responding to metabolic and cellular stress cues to promote tumor cell survival against apoptosis, whereas in normal cells, TXNDC5 acts as a friend, protecting cells against oxidative and endoplasmic reticulum stress. However, early in carcinogenesis, TXNDC5 may be downregulated due to a protective anticancer role, whereas later in tumor progression it is often upregulated. By leveraging insights from various studies on different disorders, researchers can uncover the complex molecular mechanisms that underlie TXNDC5-mediated pathologies. TXNDC5 is believed to play a regulatory role in metabolic pathways that are essential for processes such as energy production and the glucose and lipid metabolisms. By clarifying the specific functions of TXNDC5 in metabolism, researchers may discover new therapeutic strategies for managing conditions such as obesity, insulin resistance, and type 2 diabetes. Further investigation into the regulatory mechanisms of TXNDC5 within the vascular pathways may lead to the development of targeted therapies for preventing or treating cardiovascular diseases. Additionally, understanding TXNDC5’s influence on neurodegenerative processes may provide insights into the development of treatments for conditions such as Alzheimer’s disease, Parkinson’s disease, and others. Investigating the regulatory mechanisms of TXNDC5 in cancer pathways may lead to the development of targeted interventions for cancer management. Furthermore, TXNDC5 could potentially serve as a diagnostic marker for certain types of cancer, aiding in early detection and personalized treatment approaches. Overall, future research aimed at elucidating the specific regulatory mechanisms of TXNDC5 in these pathways holds promise. This will pave the way for the development of targeted interventions and personalized therapeutic approaches in precision medicine.

## Figures and Tables

**Table 1:** Genes associated with human 6p25.1p24.3 microdeletion [23].

Name	Symbol	Related Pathway	Localization
Thioredoxin domain containing 5	*TXNDC5*	Unfolded protein response	Endoplasmic reticulum
Neuritin 1	*NRN1*	Metabolism of proteins, Post-translational modification	Extracellular
Coagulation factor XIII a chain	*F13A1*	Response to elevated platelet cytosolic Ca^2+^	Extracellular
Ras responsive element binding protein 1	*RREB1*	Ras/Raf-mediated cell differentiation	Nucleus
Signal sequence receptor subunit 1	*SSR1*	Unfolded protein response	Endoplasmic reticulum
Rio kinase 1	*RIOK1*	rRNA processing in the nucleus and cytosol Processing of capped intron-containing pre-mRNA	Cytosol, nucleus
Desmoplakin	*DSP*	Signaling by Rho GTPases	Nucleus, plasma membrane
Bone morphogenetic protein 6	*BMP6*	Apoptotic pathways in synovial fibroblasts MIF-mediated glucocorticoid regulation	Extracellular
Biogenesis of lysosomal organelles complex 1 subunit 5	*BLOC1S5*	Biogenesis of lysosome-related organelles	Cytosol
Eukaryotic translation elongation factor 1 epsilon 1	*EEF1E1*	Peptide chain elongation tRNA aminoacylation	Cytosol, nucleus
Solute carrier family 35 member b3	*SLC35B3*	Biotransformation Glycosaminoglycan metabolism	Golgi apparatus
Cancer antigen 1	*CAGE1*	Retinoblastoma progression	Cytosol, nucleus
Lymphocyte antigen 86	*LY86*	MyD88 dependent cascade initiated on endosome	Extracellular
Hepatocellular carcinoma upregulated long non-coding RNA	*HULC*	Cancer progression	Ribosome

**Table 2:** Non-coding RNA interacting with TXNDC5.

Classification	Species	Disease	References
Long non-coding RNAs	LncRNA-ENST00000556926	Lung cancer	[34]
Circular RNAs	CircRNA_0078710	Hepatocellular carcinoma	[2,35]
	CircRNA-104718	Hepatocellular carcinoma	[37]
	CircRNA_0000517	Hepatocellular carcinoma	[2,36]
MicroRNAs	miRNA-431-5p	Hepatocellular carcinoma	[2,35]
	miRNA-1296-5p	Hepatocellular carcinoma	[2,36]
	miRNA-218-5p	Hepatocellular carcinoma	[37]
	miRNA-30c-5p	Hepatocellular carcinoma	[38]
	miRNA-30e-5p	Hepatocellular carcinoma	[38]
	miRNA-573	Rheumatoid arthritis	[2,39,40]
	miRNA-200b	Prostate cancer	[2,42]
	miRNA-92a	Atherosclerosis	[43]
	miRNA-124	Medulloblastoma	[41]
MicroRNA offset RNAs	moRNA-21	Breast cancer	[44]
